# Glycyrrhizic acid alleviates the meconium-induced acute lung injury in neonatal rats by inhibiting oxidative stress through mediating the Keap1/Nrf2/HO-1 signal pathway

**DOI:** 10.1080/21655979.2021.1937445

**Published:** 2021-09-09

**Authors:** Linhan Zhu, Meichen Wei, Nan Yang, Xuehua Li

**Affiliations:** Pediatric Department, Beijing Friendship Hospital, Capital Medical University, Beijing China

**Keywords:** Glycyrrhizin, meconium aspiration syndrome, oxidative stress, nrf2

## Abstract

Meconium aspiration syndrome (MAS) is a disease closely related to inflammation and oxidative stress. Glycyrrhizic acid (GA) is a triterpenoid isolated from licorice with multiple bioprotective properties. In the present study, impacts of GA against MAS rats, as well as the potential mechanism, will be investigated. MAS model was established on newborn rats, followed by the treatment of 12.5, 25, and 50 mg/kg GA. The wet/dry weight ratio of lung tissues was calculated. The production of IL-6, IL-1β, TNF-α, malonaldehyde (MDA), superoxide dismutase (SOD), glutathione (GSH) was measured using ELISA assay. HE staining was used to evaluate the pathological state of lung tissues and TUNEL assay was used to detect the apoptotic state. The protein expression of Nrf2, Keap1, HO-1, Bcl-2, Bax, and cleaved-Caspase3 was measured by Western blotting assay. The elevated W/D ratio, release of inflammatory factors, lung injury score, and apoptotic index, as well as the activated oxidative stress and suppressed Keap1/Nrf2/HO-1 pathway, in MAS rats were significantly alleviated by GA. After introducing the inhibitor of Nrf2, ML385, the protective property of GA on the pathological state, apoptotic index, and oxidative stress in MAS rats was pronouncedly abolished. Taken together, glycyrrhizin alleviated GAH in rats by suppressing Keap1/Nrf2/HO-1 signaling mediated oxidative stress.

## Introduction

**Figure:**
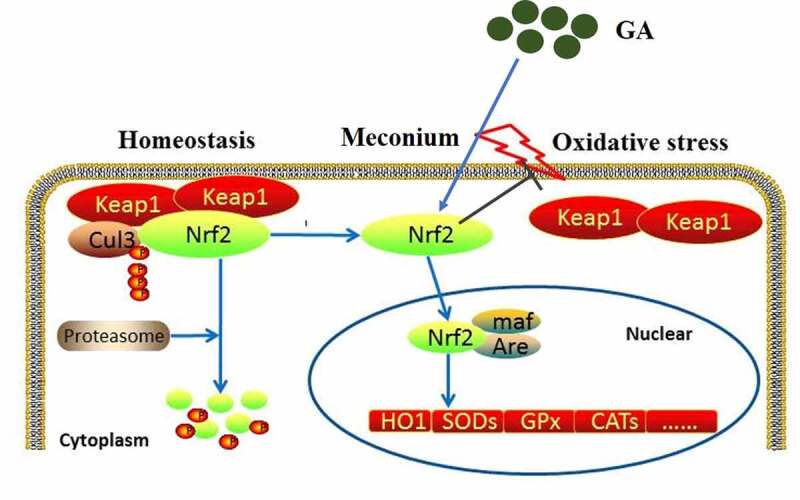

Meconium aspiration syndrome (MAS) is a typical disease observed in newborn and is reported to contribute to severe respiratory failure and even death, which is commonly developed during the perinatal and neonatal period [1]. Chemical pneumonia, airway obstruction, airway injury, and surfactant inactivation will be induced by the inhalation of meconium [2]. The pathological mechanism underlying MAS is complicated, in which the inhalation of meconium is reported to be involved [3].

Meconium is a mixture of water-soluble (bilirubin, bile acid, enzyme, etc.) and liposoluble (free fatty acids, cholesterol, triglycerides, etc.) substances, among which liposoluble materials show the most significant impacts on the lung tissues [4]. In the clinic, after the inhalation of meconium, meconium will travel into the alveoli and the viscosity of the pulmonary surfactant will be reduced and the ultrastructure of the pulmonary surfactant will be changed by the liposoluble materials in the meconium, which result in the decreased level of surfactant proteins [5,6]. As a consequence, the plasma protein leakage will be induced and the local inflammation will be developed, including the excessive production of inflammatory factors [7,8]. In addition, the release of proteolytic enzymes will be facilitated and the imbalance of oxidative stress will be triggered by the severe inflammation, which finally contributes to the injuries on lung tissues [9]. Therefore, the anti-inflammatory and anti-oxidative stress therapy is an effective method to treat MAS.

Glycyrrhizin (GL) is a triterpenoid isolated from licorice and is also named as glycyrrhizic acid (GA). As the active ingredient of licorice, extensive biological functions of GL have been widely reported, including anti-inflammatory, antiviral, and immunomodulatory properties [10]. Currently, in the clinic, GA is mainly applied for to treat acute and chronic hepatitis, rheumatoid, and skin diseases. Recent researches reported the significant functions of GA on reducing the secretion of reactive oxygen species (ROS), anticoagulation, and anti-oxidative stress [11,12].

In the present study, we suspected that GA might exert protective effects on MAS, which might be related to the regulation of oxidative stress. The present study aims to explore the potential therapeutic property for the treatment of MAS by investigating the impact of GA against meconium treated neonatal rats, as well as the inhibitory effect of GA on inflammation and oxidative stress.

## Materials and methods

**The establishment of a MAS model on rats and the grouping of the animal experiments**: After achieving the agreements from the pregnant women, the first meconium was taken from the healthy term newborns, followed by vacuum freeze drying for 24 hours. Subsequently, the meconium was grounded into powder and dissolved in the normal saline into a 40 mg/mL suspension, which was stored at −20°C to be used for the subsequent experiments. According to the method described by Zagariya [13], immature rats were anesthetized using 2% 40 mg/kg pentobarbital, followed by fixed on the operating table. After tracheotomy, trachea cannula was performed and the cannula was fixed, followed by maintaining the muscle relaxant by intraperitoneally injecting with 2 mg/kg vecuronium bromide. According to the method described previously [14], a total amount of 1 mL/kg meconium was injected into the lung of the immature rat through the central, left, and right decubitus position of the cannula within 10 min, followed by injecting 3 mL air to ensure that the normal saline or meconium entered the bronchus and alveoli evenly. An equal volume of normal saline was injected into the animals in the Sham group using the same method. The cannula was taken out from all animals 1–1.5 hours post-surgery, followed by suturing the trachea, neck muscles, and skin. The normal activity and food intake were recovered after the animals’ awake.

For the pharmacodynamic experiments, the animals were divided into five groups (n = 5): sham (normal saline, i.p., 8 days), model (normal saline, i.p., 8 days), low dose GA (12.5 mg/kg, i.p., 8 days), medium dose GA (25 mg/kg, i.p., 8 days), and high dose GA (50 mg/kg, i.p., 8 days) [15]. For the verification experiment, the animals were divided into four groups (n = 5): sham (normal saline, i.p., 8 days), model (normal saline, i.p., 8 days), GA (25 mg/kg, i.p., 8 days), and GA+ML385 (25 mg/kg GA and 30 mg/kg ML385 [16], i.p., 8 days).

**The detection of the lung wet-dry weight (W/D) ratio**: The lung tissues were extracted, followed by rinsing the surface blood and residual tissues using normal saline. After blotting up the liquid on the surface, the left-lung tissue was isolated by cutting on the bifurcation of the trachea, which was weighed using the electronic scales. Subsequently, the left-lung tissue was died in the 65°C ovens for 48 hours, followed by weighing using the electronic scales. The lung W/D ratio was calculated according to the weights. W/D ratio is reported to be an important marker to evaluate the degree of lung injury, which is widely used in the animal experiments of acute lung injury [17,18].

**HE staining on the lung tissues**: The right-lung tissues of each animal were isolated and fixed in 10% formalin, followed by embedded with paraffin. Subsequently, 4 μm sections were cut on the embedded tissues and washed with PBS buffer 3–5 times, followed by incubated with the hematoxylin dye for 5 min. After incubating in the acid and 1 min incubation in the ammonia solution, respectively, the sections were stained with eosin dye for 2 min, which were further soaked in xylene for 8 min. Lastly, after sealing with neutral gum, the tissues were observed using the inverted microscope (Bato Instrument, Shanghai, China).

The pathological scores were evaluated according to the standards described previously [19]: (1) The infiltration range of leukocytes in the lung tissues: 0 = 0%; 1 = 0–25%; 2 = 25%-50%; 3 = 50%-75%; 4 = 75%-100%; (2) The amounts of leukocytes in the alveolar space: 0 = none; 1 = rare leukocytes; 2 = great amounts of leukocytes; 3 = filled with leukocytes; 4 = filled with outspread leukocytes; (3) The amounts of alveolar exudates, including cellulose, hyaline membrane, edema fluid, and meconium: 0 = no exudate; 1 = rare exudate; 2 = clearly visible exudate; 3 = filled with exudate; 4 = filled with exudate. Five fields of views were selected for each section to calculate the mean pathological score.

**TUNEL assay**: As described previously [20], after fixing the lung tissues in 4% paraformaldehyde solution for 6 h, the tissues were embedded in paraffin followed by stored in 30% sucrose solution at 4°C for 3 days. Then, 18 μm sections were cut on the embedded tissues, which were further incubated with TUNEL solution (Elabscience, Wuhan, China) at 37°C for 1 h in the dark. After three washes, DAB reagent was added to conduct the chromogenic experiment and the images were taken using the optical microscope (Bato Instrument, Shanghai, China) for the evaluation of the apoptotic state in the lung tissues. The apoptotic index was calculated as TUNEL^+^ cells/Total cells.

**The determination of MDA and SOD concentration**: The level of MDA, GSH and SOD in the samples was measured using the commercial ELISA kit (eBioscience, California, USA) in terms of the instruction.

**ELISA assay**: The concentration of MPO, IL-6, TNF-α, and IL-1β in the homogenate of lung tissues was detected by ELISA assay (Abcam, Cambridge, UK) in terms of the instruction. Finally, the absorbance at 450 nm of the samples was detected with a microplate spectrophotometer (HACH, Colorado, USA).

**Western blotting assay**: As described previously [21], following obtaining proteins from the lung tissues using the lysis solution (Biorbyt, Cambridge, UK), BCA kit (Biorbyt, Cambridge, UK) was used to quantify the isolated proteins and the proteins were loaded and separated by the 12% SDS-PAGE gel, followed by transferred to the PVDF membrane (Merck, New Jersey, USA) and the membrane was further incubated in the 5% skim milk, followed by incubating with the primary antibodies against Nrf2 (1:800, Biorbyt, Cambridge, UK), Keap1 (1:800, Biorbyt, Cambridge, UK), HO-1 (1:800, Biorbyt, Cambridge, UK), Bcl-2 (1:800, Biorbyt, Cambridge, UK), Bax (1:800, Biorbyt, Cambridge, UK), cleaved-Caspase3 (1:800, Biorbyt, Cambridge, UK), or GAPDH (1:800, Biorbyt, Cambridge, UK) at 4°C overnight. Then, the membrane was incubated with secondary antibody (1:4000, Biorbyt, Cambridge, UK) for 2 h. Lastly, following exposure, the bands were analyzed using the Image J software (Bio-Rad, California, USA) with GAPDH as the loading control.

**Statistical Analysis**: The data in the present study were expressed as mean ± SD, which were analyzed using one-way ANOVA for the test of differences between groups. For Dunnett’s T3 was used to analyze the data with unequal variances. P < 0.05 was considered statistically significant.

## Results

We suspected that GA might exert protective effects on MAS by inhibiting oxidative stress. The purpose of the present study is to explore the potential therapeutic property for the treatment of MAS by investigating the impact of GA on meconium treated neonatal rats, as well as the inhibitory effect of GA on inflammation and oxidative stress. We established the MAS rat model and treated the model animals with different dosages of GA to evaluate the therapeutic effects of GA on MAS rats, followed by detecting the changes of oxidative stress and the Nrf2 pathway. Subsequently, the MAS rats were co-administered with GA and the inhibitor of Nrf2 pathway, ML385, to verify that the therapeutic effects of GA were related to the activation of Nrf2 pathway.

**Figure 1.**
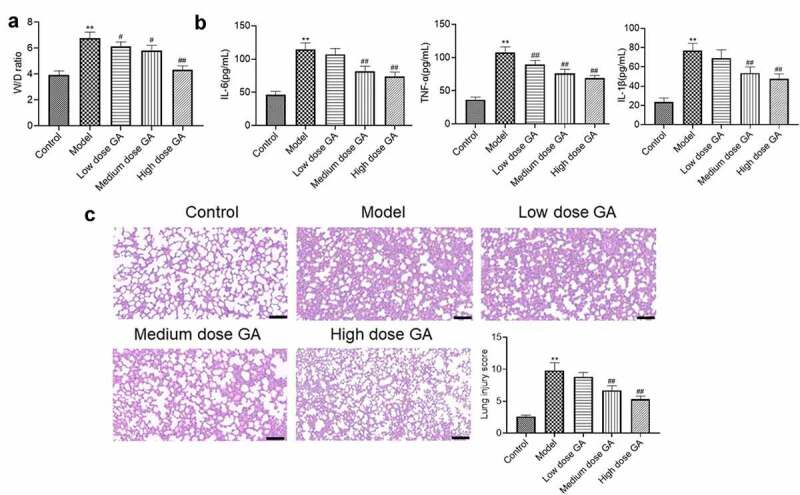
The pathological state of MAS rats was significantly alleviated by GA. A. The W/D ratio of lung tissues was calculated. B. The concentration of IL-6, IL-1β, and TNF-α was detected by ELISA assay. C. HE staining was used to detect the pathological state of lung tissues (**p < 0.01 vs. Control, #p < 0.05 vs. Model, ##p < 0.01 vs. Model)

**GA alleviated the symptom of MAS in newborn rats**: After treated with different dosages of GA, the lung tissues were isolated for the pathological analysis. Compared to control (Figure 1a), the average W/D ratio was significantly elevated from 3.91 to 6.75 in the model group, which was dramatically suppressed to 6.12, 5.79, and 4.34 by the treatment of 12.5, 25, and 50 mg/kg GA, respectively (**p < 0.01 vs. Control, #p < 0.05 vs. Model, ##p < 0.01 vs. Model).

We further measured the production of inflammatory factors in the lung tissues, the results of which are shown in Figure 1b. Compared to control, the release of IL-6 in the model group was significantly promoted from 46.2 pg/mL to 114.6 pg/mL, which was further decreased to 107.2, 81.4, and 74.2 pg/mL by the administration of 12.5, 25, and 50 mg/kg GA, respectively (**p < 0.01 vs. Control, ##p < 0.01 vs. Model). The production of TNF-α in the control, model, low dose GA, medium dose GA, and high dose GA groups was 36.7, 107.5, 89.4, 76.3, and 68.9 pg/mL, respectively (**p < 0.01 vs. Control, ##p < 0.01 vs. Model). Lastly, compared to control, the production of IL-1β was dramatically elevated from 23.7 pg/mL to 76.8 pg/mL in the model group, which was further suppressed to 69.2, 53.7, and 47.5 pg/mL by the treatment of 12.5, 25, and 50 mg/kg GA, respectively (**p < 0.01 vs. Control, ##p < 0.01 vs. Model).

Subsequently, HE staining was performed on the lung tissues and the lung injury scores were determined according to the standards described in the part of methods. Compared to control (Figure 1c), the average lung injury score was significantly promoted from 2.6 to 9.8, which was greatly declined to 8.8, 6.7, and 5.3 by the administration of 12.5, 25, and 50 mg/kg GA, respectively (**p < 0.01 vs. Control, ##p < 0.01 vs. Model). These results revealed that the MAS-like symptoms observed in newborn MAS rats were dramatically mitigated by GA.

**Figure 2.**
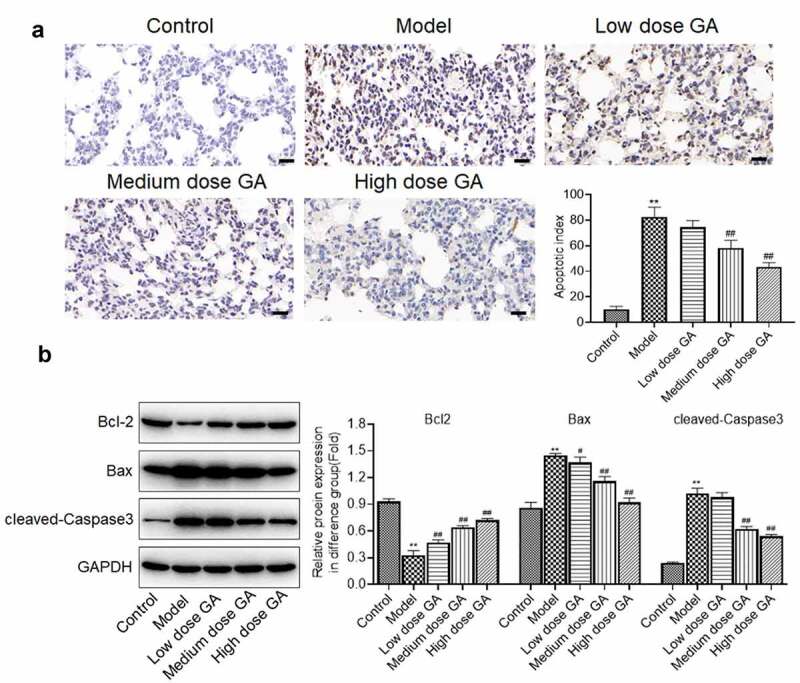
The apoptotic state of lung tissues from MAS rats was significantly mitigated by GA. A. The apoptotic index of lung tissues was evaluated by TUNEL assay. B. The expression of Bcl-2, Bax, and cleaved-Caspase-3 was determined by Western blotting assay (**p < 0.01 vs. Control, #p < 0.05 vs. Model, ##p < 0.01 vs. Model)

**GA ameliorated the apoptotic state in the lung tissues of MAS newborn rats**: The apoptotic state in the lung tissues isolated from each animal was further detected. The apoptotic index (Figure 2a) in the model group was greatly higher than that in the control group, which was greatly decreased in the medium dose GA and high dose GA group (**p < 0.01 vs. Control, ##p < 0.01 vs. Model). Compared to control, the expression of Bcl-2 was dramatically suppressed and the expression of Bax and cleaved-Caspase 3 was greatly elevated in the model group, which were dramatically reversed by the treatment of different dosages of GA (**p < 0.01 vs. Control, #p < 0.05 vs. Model, ##p < 0.01 vs. Model). These data revealed that the apoptotic state in the lung tissues of MAS newborn rats was significantly ameliorated by GA.

**Figure 3.**
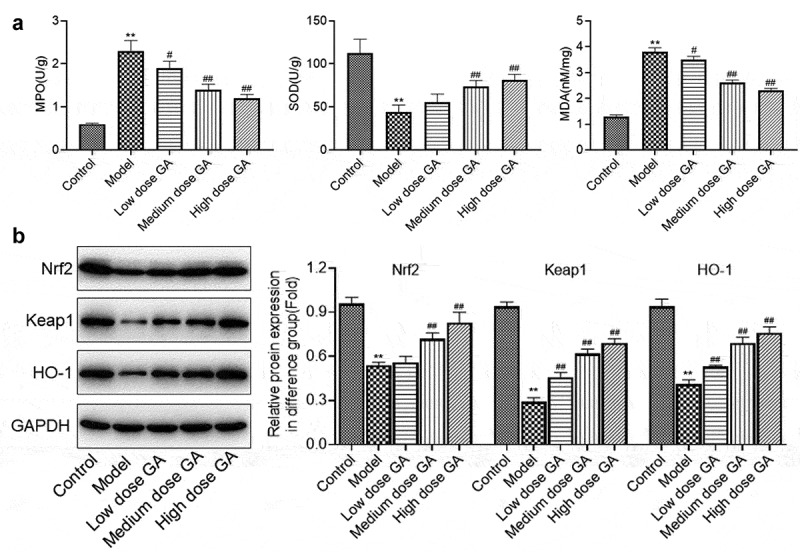
The oxidative stress in WAS rats was dramatically suppressed by GA. A. The concentration of MDA, SOD, and GSH was determined by ELISA assay. B. The expression of Nrf2, Keap1, and HO-1 was determined by Western blotting assay (**p < 0.01 vs. Control, #p < 0.05 vs. Model, ##p < 0.01 vs. Model)

**GA mitigated the oxidative stress in lung tissues of MAS newborn rats**: The effects of GA on oxidative stress were subsequently investigated. As shown in Figure 3a, the secretion of MPO was significantly elevated from 0.6 U/g to 2.3 U/g, which was greatly declined to 1.9, 1.4, and 1.2 U/g by the treatment of 12.5, 25, and 50 mg/kg GA, respectively (**p < 0.01 vs. Control, #p < 0.05 vs. Model, ##p < 0.01 vs. Model). The activity of SOD was dramatically higher in the model group than that in the control group, which declined greatly in the medium dose GA and high dose GA group (**p < 0.01 vs. Control, ##p < 0.01 vs. Model). Lastly, the concentration of MDA in the control, model, low dose GA, medium dose GA, and high dose GA groups was 1.3, 3.8, 3.5, 2.6, and 2.3 nM/mg, respectively (**p < 0.01 vs. Control, #p < 0.05 vs. Model, ##p < 0.01 vs. Model). As shown in Figure 3b, Nrf2, Keap1, and HO-1 were dramatically downregulated in the model group, which were significantly upregulated in the GA-treated groups (**p < 0.01 vs. Control, ##p < 0.01 vs. Model). These data revealed that the oxidative stress in MAS newborn rats was significantly mitigated by GA.

**Figure 4.**
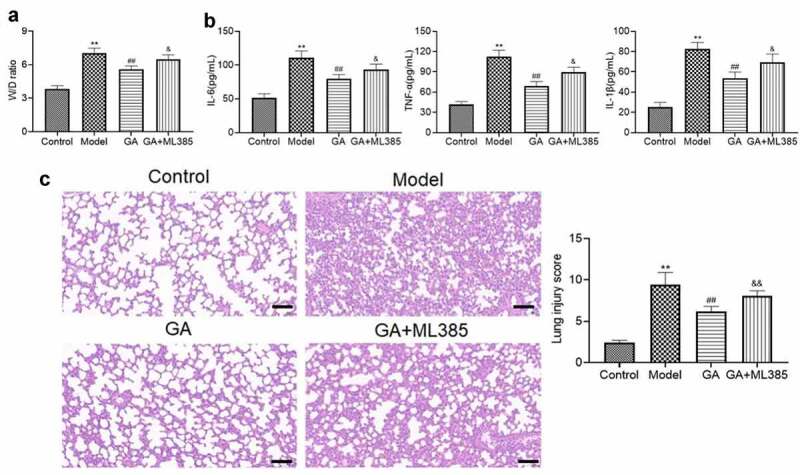
The protective effect of GA on the pathology of WAS tissues was abolished by ML385. A. The W/D ratio of lung tissues was calculated. B. The concentration of IL-6, IL-1β, and TNF-α was detected by ELISA assay. C. HE staining was used to detect the pathological state of lung tissues (**p < 0.01 vs. Control, ##p < 0.01 vs. Model, ^&^p < 0.05 vs. GA, ^&&^p < 0.01 vs. GA)

**The protective effects of GA on MAS rats were abolished by ML385**: To verify the involvement of Keap1/Nrf2/HO-1 signaling in the mechanism underlying the therapeutic effect of GA, MAS animals were co-administered with both GA and ML385, an inhibitor of Keap1/Nrf2/HO-1 signal pathway. As shown in Figure 4a, the elevated W/D ratio in the model group was greatly suppressed by the treatment of GA, which was further greatly reversed by the co-treatment of ML385 (**p < 0.01 vs. Control, ##p < 0.01 vs. Model, ^&^p < 0.05 vs. GA).

The concentration of inflammatory factors was measured (Figure 4b). The release of IL-6 was dramatically elevated from 51.2 pg/mL to 110.8 pg/mL, which was greatly suppressed to 79.5 pg/mL by the treatment of GA. Compared to the GA group, the release of IL-6 was dramatically reversed to 93.1 pg/mL by the treatment of GA in the presence of ML385. In addition, the concentration of TNF-α in the control, model, GA, and GA+ML385 groups was 41.7, 112.5, 69.3, and 89.4 pg/mL, respectively. Lastly, the production of IL-1β was pronouncedly elevated from 25.4 pg/mL to 82.5 pg/mL in the model group and was further inhibited to 53.7 pg/mL by the administration of GA, which was greatly reversed to 69.2 pg/mL by the co-administration of GA and ML385 (**p < 0.01 vs. Control, ##p < 0.01 vs. Model, ^&^p < 0.05 vs. GA).

Subsequently, the pathology of lung tissues was measured using HE staining. As shown in Figure 4c, the average lung injury score was significantly elevated from 2.4 to 9.4 in the model group and was further declined to 6.2 in the GA treated animals, which was greatly reversed to 8.1 by the co-administration of GA and ML385 (**p < 0.01 vs. Control, ##p < 0.01 vs. Model, ^&&^p < 0.01 vs. GA). These data revealed that the protective property of GA against MAS rats was abolished by the blockage of Keap1/Nrf2/HO-1 signaling.

**Figure 5.**
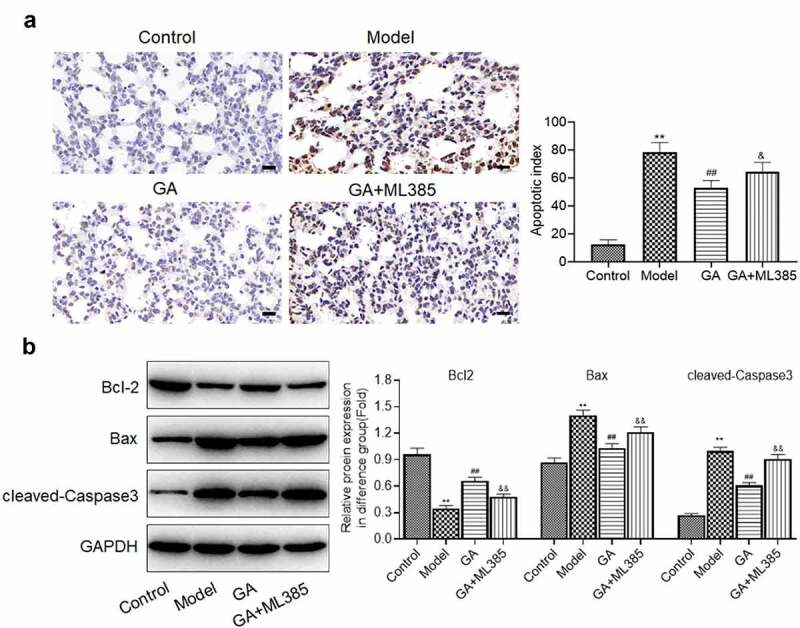
The protective property of GA on the apoptotic state of WAS tissues was abolished by ML385. A. The apoptotic index of lung tissues was evaluated by TUNEL assay. B. The expression level of Bcl-2, Bax, and cleaved-Caspase-3 was determined by Western blotting assay (**p < 0.01 vs. Control, ##p < 0.01 vs. Model, ^&^p < 0.05 vs. GA, ^&&^p < 0.01 vs. GA)

**ML385 abolished the protective effect of GA on the apoptosis in MAS rats**: We further measured the apoptotic state in the lung tissues isolated from each animal. The elevated apoptotic index in the model group (Figure 5a) was greatly decreased in the GA group, which was dramatically reversed by the co-treatment of GA and ML385 (**p < 0.01 vs. Control, ##p < 0.01 vs. Model, ^&^p < 0.05 vs. GA). In addition, the declined expression of Bcl-2 in the model group (Figure 5b) was pronouncedly promoted by the treatment of GA, which was greatly suppressed in the GA+ML385 group. The upregulated Bax and cleaved-Caspase 3 in the model group were dramatically downregulated in the GA group, which were greatly reversed in the GA+ML385 group (**p < 0.01 vs. Control, ##p < 0.01 vs. Model, ^&&^p < 0.01 vs. GA). These data revealed that the protective property of GA on the apoptosis in MAS rats was significantly abolished by the blockage of Keap1/Nrf2/HO-1 signaling.

**Figure 6.**
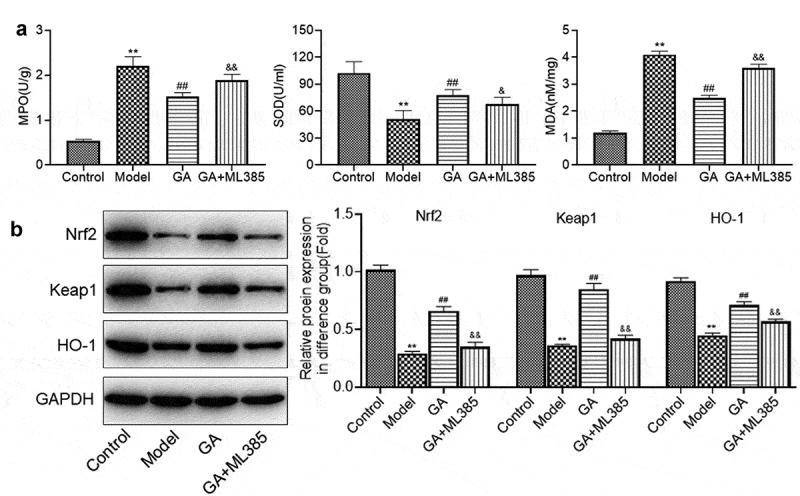
The protective effect of GA on oxidative stress of WAS tissues was abolished by ML385. A. The concentration of MDA, SOD, and GSH was determined by ELISA assay. B. The expression of Nrf2, Keap1, and HO-1 was determined by Western blotting assay (**p < 0.01 vs. Control, ##p < 0.01 vs. Model, ^&^p < 0.05 vs. GA, ^&&^p < 0.01 vs. GA)

**ML385 abolished the protective effect of GA on the oxidative stress in MAS rats**: The production of MPO (Figure 6a) was significantly elevated from 0.53 U/g to 2.2 U/g in the model group and was further decreased to 1.52 by the treatment of GA, which was greatly reversed to 1.89 in the GA+ML385 group (**p < 0.01 vs. Control, ##p < 0.01 vs. Model, ^&&^p < 0.01 vs. GA). The activity of SOD in the control, model, GA, and GA+ML385 groups was 102.4, 51.4, 78.1, and 67.8 U/mL, respectively (**p < 0.01 vs. Control, ##p < 0.01 vs. Model, ^&^p < 0.05 vs. GA). The promoted concentration of MDA in the model group was pronouncedly suppressed in the GA group, which was greatly reversed in the GA+ML385 group (**p < 0.01 vs. Control, ##p < 0.01 vs. Model, ^&&^p < 0.01 vs. GA). As shown in Figure 6b, the downregulated Nrf2, Keap1, and HO-1 in the model group were dramatically upregulated in the GA group, which was greatly reversed in the GA+ML385 group (**p < 0.01 vs. Control, ##p < 0.01 vs. Model, ^&&^p < 0.01 vs. GA). These data indicated that protective property of GA on the oxidative stress in MAS rats was significantly abolished by the blockage of Keap1/Nrf2/HO-1 signal pathway.

## Discussion

Currently, the pathological mechanism underlying the development of MAS remains unclear. It is reported that imbalance of cytokines, anoxia, dysfunction of endothelial cells, infection, oxidative stress, and the cell apoptosis are important factors involved in the development and processing of MAS [22]. In addition, severe inflammation is triggered by inactivation of surfactants, infiltration of neutrophils and leukocytes, and excessive released inflammatory factors [23]. Large-scale cell death in the lung tissues can be induced by the severe inflammation after the stimulation of meconium. It is reported that 8 hours post the treatment of meconium, approximately 70% apoptotic bodies are found in the airway epithelial cells and the apoptosis is also found in the alveolar epithelial cells [24,25]. In the present study, a MAS model was successfully established on rats, which was verified by the symptoms, such as increased W/D ratio, elevated production of inflammatory factors, and aggravated apoptotic state in the lung tissues, which was consistent with the pathological changes described previously [26,27]. In addition, according to the results of HE staining, significant infiltration of leukocytes and large amounts of alveolar exudates were observed in the lung tissues from MAS rats, which corresponded to the description by Turhan [7]. After the treatment of different dosages of GA, the elevated W/D ratio, severe inflammation and apoptotic state, significant infiltration of leukocytes, and the accumulated alveolar exudates were dramatically ameliorated, revealing a promising therapeutic property of GA on MAS rats.

Oxidative stress is recently reported to be closely associated with the pathogenesis of MAS [28]. Under the normal physiological state, the cellular oxidative level is maintained within a dynamically controlled range by the balance between electronic receptor and the donor [29]. Oxidative stress is defined as a peroxidative state resulted from the unbalance of intracellular REDOX induced by the excessive accumulated reactive oxygen species (ROS) [30]. The excessive ROS will react with such small molecules as proteins, membrane lipid, and nucleic acids, which contributes to the production of peroxides. The cellular structure will be disrupted by the produced peroxides and the normal cellular physiological function will be impacted, which further induces the changes of related signal pathways and finally the cell death [31]. The elevated concentrations of MDA and GSH, as well as the declined activity of SOD, are reported to be important biomarkers of the development of oxidative stress [32]. In this study, we observed that oxidative stress was significantly activated in the lung tissues isolated from MAS rats, which was consistent with the description in previous reports [28]. After the treatment of medium and high dosage of GA, the state of oxidative stress in the lung tissues was significantly alleviated, indicating that GA might exert promising therapeutic effects on MAS by inhibiting oxidative stress in the lung tissues.

Keap1/Nrf2/HO-1 signaling is an important anti-oxidative pathway that regulates the balance between the oxidation system and anti-oxidation system [33]. Under the normal physiological state, Nrf2 and Keap1 are located in the cytoplasm. However, when oxidative stress is activated, protein kinases will be activated to induce the phosphorylation of Nrf2, which further disassociates with Keap1 and is transferred into the nucleus [34]. The entered Nrf2 combines with the antioxidant reaction element (ARE) to initiate the transcription of antioxidation proteins, such as heme oxygenase-1 (HO-1) [35]. In the present study, the Keap1/Nrf2/HO-1 signaling was found to be significantly suppressed in the lung tissues of MAS rats, indicating that the anti-oxidative system was inhibited. After the treatment of GA, the Keap1/Nrf2/HO-1 signaling was activated, accompanied by the alleviation of oxidative stress. We suspected that GA might mitigate oxidative stress in the lung tissues by activating the Keap1/Nrf2/HO-1 pathway, which was further verified by the introduction of an inhibitor of Nrf2, ML385.

Currently, the main therapeutic drugs for the treatment of MAS include antibiotics [36], inhalation of nitric oxide (NO) [37], glucocorticoid [4], and recombinant human superoxide dismutase [9]. However, these therapies mainly aim to eliminate inflammation during the development of MAS, with different degrees of side effects. In the present study, the potential therapeutic property of a herbal medicine against MAS was explored for the first time, which might be a promising anti-MAS agent with clear mechanism and less side effects. However, the conclusion needs to be verified by more preclinical and clinical data.

In our future work, the direct target of GA will be investigated in the in vitro model, such as meconium treated alveolar epithelial cells, by screening the differentiated genes using the technology of gene microarrays. The binding between GA and the screened target will be verified using the immunoprecipitation technology and the function of the screened target protein will be confirmed in the MAS animal model. These data will help better to understand the therapeutic properties of GA against MAS.

## Conclusion

Our data revealed that GA alleviated the meconium-induced acute lung injury in neonatal rats by inhibiting oxidative stress via mediating the Keap1/Nrf2/HO-1 signal pathway.

## Data Availability

The data used to support the findings of this study are available from the corresponding author upon request.

## Research Highlights

1. Glycyrrhizin alleviates MAS.
2. Glycyrrhizin ameliorates oxidative stress in MAS rats.
3. Glycyrrhizin mitigates oxidative stress by regulating Nrf2 pathway.

- Glycyrrhizin alleviates the meconium-induced acute lung injury in newborn rats;
- Glycyrrhizin suppresses oxidative stress and inflammation in the lung tissues;
- Glycyrrhizin alleviates oxidative stress via mediating the Keap1/Nrf2/HO-1 signal pathway.
